# 

**Published:** 1845-01

**Authors:** 


### CONTENTS

### OF NO. I.

### ORIGINAL COMMUNICATIONS.

### ESSAYS AND CASES.

Art. I.—Remarks on Mustard Poultices, applied extensively to the surface. By William O. Baldwin, M.D., of Montgomery, Ala. - - - - - 1
Art. II.—A Case of Emphysema By E. W. Harris, M. D., of Cape Girardeau county, Mo. - - - - 11
Art. III.—Observations on Erysipelatous Fever, as it occurred in Memphis, Tenn., in April and May, 1844, with a report of 8 cases. By Lewis Shanks, M.D. - 12

### REVIEWS.

Art. IV.—Lectures on the more important Diseases of the Thoracic and Abdominal Viscera; and on Eruptive Fevers, Hæmorrhages, and Dropsies; and on Gout and Rheumatism. Delivered in the University of Pennsylvania. By N. Chapman, M.D., Professor of the Theory and Practice of Medicine, &c. - - 24
Art. V.—Ethics of Medicine: An Anniversary Address delivered the 3d of April, 1844, before the Medico-Chirurgical Society of Louisiana. By Thomas M. Logan, M.D. Published by the Society. - - - 47
The Claims of Religion on Medical Men: A Discourse, delivered in the Tenth Presbyterian Church, Philadelphia, on Sunday evening, November 24, 1844. By B. A. Boardman, Pastor of the Church. - - - 47
Art. VI.—The Cyclopædia of Practical Medicine. Edited by John Forbes, M.D., F.R.S., Alex. Tweedie,

M.D., F.R.S., and John Conolly, M.D. Revised, with additions; by Robley Dunglison, M.D. - - 58
Art. VII.—Lectures on the Theory and Practice of Physic. By John Bell, M.D., and by Wm. Stokes, M.D. - 59

SELECTIONS FROM AMERICAN AND FOREIGN JOURNALS

Lithotomy, - - - - - - - - - - - 61
Decomposition of Tinct. Opii by Ammonia, - - - 62
Scarification of the Gums—Reasons for it, - - - - 63
Caution in giving Albumen as an Antidote, - - - - 64
Alcoholic Odor of the Fluid in the Ventricles of the Brain, - 65
Malpractice, - - - - - - - - - - 65
On the Possibility of Supporting Life on a Vegetable Diet, - 68
Hygienic condition of Towns, - - - - - - - 69
Dr. C. J. B. Williams on Constipation, - - - - 74
Mania a Potu, - - - - - - - - - 80
Early Puberty in Greece, - - - - - - - 81
Organ of Calculation, - - - - - - - - 81
Prestat's Adhesive Plaster, - - - - - - - 82
On the Employment of Castor Seeds, - - - - - 82
Ovarian Disease, - - - - - - - 83
Bronchocele, - - - - - - - - - 83
Reunion of a Divided Nerve, - - - - - - 83
Medical Charity Fund of Lower Canada, - - - - 84

ORIGINAL INTELLIGENCE.

Medical Institute of Louisville, - - - - - . - 85
Rutherford county Medical Society, - - - - - 87
Introductory Lectures, - - - - - - - - 88
Southern Medical and Surgical Journal, - - - - 91
Report of the Physicians to the Kentucky Penitentiary, - - 92
Louisville Summer School of Medicine, - - - - 92

